# On our rapidly shrinking capacity to comply with the planetary boundaries on climate change

**DOI:** 10.1038/srep42061

**Published:** 2017-02-07

**Authors:** Jean-Denis Mathias, John M. Anderies, Marco A. Janssen

**Affiliations:** 1 IRSTEA, UR LISC, 9 avenue des landais, 63170 Aubière, France; 2 School of Sustainability, Arizona State University, Tempe, United States; 3 School of Human Evolution and Social Change, Arizona State University, Tempe, United States; 4 Center for Behavior, Institutions and the Environment, Arizona State University, Tempe, United States

## Abstract

The planetary boundary framework constitutes an opportunity for decision makers to define climate policy through the lens of adaptive governance. Here, we use the DICE model to analyze the set of adaptive climate policies that comply with the two planetary boundaries related to climate change: (1) staying below a CO_2_ concentration of 550 ppm until 2100 and (2) returning to 350 ppm in 2100. Our results enable decision makers to assess the following milestones: (1) a minimum of 33% reduction of CO_2_ emissions by 2055 in order to stay below 550 ppm by 2100 (this milestone goes up to 46% in the case of delayed policies); and (2) carbon neutrality and the effective implementation of innovative geoengineering technologies (10% negative emissions) before 2060 in order to return to 350 ppm in 2100, under the assumption of getting out of the baseline scenario without delay. Finally, we emphasize the need to use adaptive path-based approach instead of single point target for climate policy design.

Nine planetary boundaries have recently been defined^1^,^2^ within which we expect that humanity and our societies can operate safely. One of these planetary boundaries addresses the issue of climate change through two thresholds based on the CO_2_ concentration in the atmosphere^1^,^2^ (Fig. 1) instead of thresholds based on temperature increase. Although temperature increase is directly connected to CO_2_ concentration in the atmosphere and radiative forcing, this relationship is quite uncertain due to, for example, slow feedbacks such as the decrease of ice cover or changes in global vegetation distribution^1^. Moreover, reducing atmospheric temperatures alone will not reverse ocean acidification and the associated biodiversity loss^3^,^4^. Based on these considerations, the planetary boundary on climate change is framed in terms of atmospheric CO_2_ concentrations rather than temperature. Experts^1^,^2^ suggest that “the planet was largely ice free until atmospheric CO_2_ concentrations fell to 450 ppm (±100 ppm), indicating a danger zone when concentrations of CO_2_ rise within the range of 350–550 ppm”^5^. This suggests that there are two thresholds of interest: 350 ppm under which the system is considered safe and 550 ppm above which there is a consensus about catastrophic effects in terms of loss of polar ice sheets or freshwater supplies^5^.

Despite some criticisms^6^,^7^, the planetary boundary framework is valuable for designing climate policy through the lens of adaptive governance in order to keep the earth within a safe operating space^8^. As such, we seek to analyze the characteristics of pathways that comply with planetary climate boundaries and to extract milestones for their integration into future climate policy design. In terms of future pathways, the fifth IPCC’s report (AR5) shows that there is a variety of options for curbing climate change^9^, some of which will keep the system within the safe operating space. However, the more we advance in time, the more this variety of options erodes, particularly as governments struggle with a global agreement on the reduction of CO_2_ emissions^10^, yielding a delicate balance between the policy lag for reducing greenhouse gas emissions (it is not possible to immediately reduce all CO_2_ emissions) and the lag of global warming (even without additional greenhouse gases from 2000 to 2100, the atmosphere temperature may increase by 0.6 °C^11^). To evaluate different policy options and their impact on such climate lags, both the IPCC and policy-makers use integrated assessment models (IAMs) to aid in their decision-making process^12^,^13^. Many IAMs are used for optimizing climate policy by maximizing the discounted sum of utilities over the next hundred years^14^,^15^ or so despite the fact that these optimal trajectories remain contestable because of parameter uncertainties in the long term^16^,^17^. In addition, reality has closely tracked the baseline scenario for the past 20 years^18^ suggesting that these optimal trajectories are not credible. The approach outlined here offers an alternative view. Based on the planetary boundary framework, rather than computing the optimal policy timeline for the coming decades, we aim at assessing the full set of sustainable adaptive pathways that enable the earth to stay within the safe operating space in the face of considerable uncertainty. More specifically, we seek to identify critical boundaries in terms of time (i.e., how long can we postpone an effective global agreement?) and in terms of policy (i.e., what are the minimal policy measures we can take to prevent the crossing of the planetary climate boundaries?). An important message that emerges from the approach we take here is the need to take an adaptive, path-based approach to climate policy design rather than point targets which are meaningless in the face of social and political delays.

## Methods

### A set of adaptive pathways

Scholars and climate policy makers alike are continuously grappling with how to integrate uncertainties into their decision-making processes^19^,^20^,^21^,^22^. In this context, decision makers need tools that enable flexible and adaptive options^23^ for complying with their objectives, especially for climate change that is subjected to a diversity of uncertainties^24^. Such an adaptive approach yields a range of acceptable outcomes while avoiding irreversible negative effects, defined here by remaining within the planetary boundaries on climate change. There are a number of different approaches to generating adaptive policies and satisfying constraints (e.g. planetary boundaries) in order to stay within a given set (e.g. safe operating space). One example, the tolerable windows approach (TWA)^25^ mathematically considers all possible options from a given initial state. Unlike TWA which works forward in time, we use a similar approach, viability theory^26^,^27^, that works backward in time. Viability theory considers all initial states and computes policy options according to each states of the system at each time step. The resulting policies are adaptive to the world’s states, which is relevant for defining climate policies where the future remains uncertain. Viability theory works backwards from specified constraints and enables us to mathematically find all policy options that comply with these constraints from a given set of initial conditions. That is, if we want to hit a target in 2050 starting from where we are today, what are all the feasible policy options that will get us there? This method provides traction for managing environmental systems within an acceptable range^28^,^29^ which, here, is based on the planetary boundaries^1^,^2^. The remainder of the paper focuses on using viability theory to provide some new insights regarding the trade-offs between policy lags and climate in terms of adaptive policies aimed at remaining within the climate change planetary boundary.

### The DICE model

Among IAMs, the DICE model^30^,^31^,^32^ (2013R) is one of the most commonly used by scholars and national agencies (e.g. the US Environmental Protection Agency). This model has been broadly used for optimizing the emission reduction rate of CO_2_ and reinvestment of the gross world product (GWP) according to different constraints and criteria. In what follows, we use the basic processes of the DICE model to model the dynamics of 6 state variables with a time step of 5 years (see SI for detailed equations): temperatures in the atmosphere (*T*_*AT*_(*t*)) and in the ocean (*T*_*LO*_(*t*)), a carbon stock in the atmosphere *M*_*AT*_(*t*), a carbon stock in the ocean *M*_*UP*_(*t*), the world capital stock *K*(*t*), and the CO_2_ emission control rate *μ*(*t*). Policy options rely on two controls: the fraction, of the gross world product (GWP) that is reinvested in the world capital stock, *I*(*t*), as well as the relative increase, *α*_*μ*_(*t*), (every 5 years) of the CO_2_ emission reduction rate, *μ*(*t*), defined as follows:


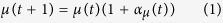


With *μ*(*t*) in [0; 1] and *α*_*μ*_(*t*) in [0; 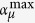]. The value of *α*_*μ*_(*t*) is equal to 0 when *μ*(*t*) = 1. The value of 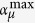 constitutes the maximum capacity to reduce CO_2_ emissions. The mean value of *α*_*μ*_(*t*) is around 0.4 for the temperature-limited scenario. In order to have enough flexibility around this mean value, we fix the reference value of 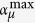 to 1, *i.e.* the value may double every 5 years. Note that this value is specifically analyzed and discussed (see Fig. 2) in the results section. The time horizon is 2100, with *t*_0_ = 2010. The investment (savings) rate *I*(*t*) ranges between the minimum (0.2366) and maximum (0.2592) values found in the baseline and the limited-temperature scenarios in the DICE model.

### Viability theory

Viability theory^33^ aims to preserve some set of desired properties (denoted by *K*) of a dynamical system. In the viability framework, controls are explicit expressed in the dynamics and not fixed beforehand. Instead, the goal is to find relevant management strategies (not necessarily “optimal” according to a certain criterion) that will maintain the system within its state constraint set *K*. In discrete time, this means that at each time step, there is a set of possible controls that one must choose from. Defining the state of the system as a vector ***x***(***t***) at time *t* and a vector ***u***(***t***) **∈** ***U*** as possible controls, a typical controlled discrete-time dynamical system can be written in general as:


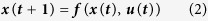


One objective of the viability theory is to determine the present states in *K* for which future system states can be kept in *K* – here the planetary boundaries on climate change – for the dynamics defined by ***f***(***x***(***t***), ***u***(***t***)) until *T* (a finite horizon) This objective is achieved through management strategies ***u***(***t***) associated with any date *t* and any state *x*. In the DICE model, these controls are ***α***_***μ***_(***t***) and *I*(*t*), i.e. ***u***(***t***) = [***α***_***μ***_(***t***); ***I***(***t***)]. The set of all the states for which there is a control strategy such that the earth state can be maintained at all times in the planning horizon *T* inside the planetary boundary is called the “viability set” or the “viability kernel” ***Viab***(***K**, **T***). The viability kernel ***Viab***(***K**, **T***) is composed of all initial states of systems for which there is at least a sequence of action policies ***u***(***t***) which influences the evolution of ***x***(***t***) at time *t* and allows the system to stay in this same viability kernel into the future. In discrete time, it can be formally defined as the set of initial states for which there exists a trajectory that does not leave *K* until the finite horizon *T*:


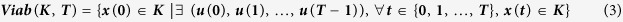


It is important to note that in a sequential decision process, the mathematical term “initial states” refers to “present state”, i.e. “now”. That is, the “present state” is the initial state of any decision process into the future. Within the viability kernel, the system can be maintained in a desirable state indefinitely for so long as it is not disturbed - it is, by definition, the set of all the viable states. The computation of a viability kernel also yields the set of controls that maintain the system, the so-called the viable controls.

Some reflection should make it clear that calculating *Viab*(*K, T*) and the viable controls is quite challenging. For our calculations, we have used Saint-Pierre’s algorithm^33^ (see ref. 34 for algorithm implementation). In our case, we have only one constraint (on CO_2_ concentrations), the other dimensions being not constrained. However, given the computational challenges associated with finding all viable states and controls, we have to limit the range of our analysis. We chose a range delimited by the minimum and maximum values for each of the six state variables of the baseline and temperature-limited scenarios. From the range of analysis, it is then necessary to define a range for the actual viability computations by finding all states reachable from the analysis range. Table S2 presents both ranges and explains the relationships between them. Next, it is important to note that we cannot consider all possible values of the state and control variables within these ranges – this is not practical (it would take an infinite amount computational time). We must thus discretize the state space. Thus, for each control and each state variable, we consider 11 uniformly distributed values with a time step of 5 years. As a result, we consider nearly 11^6^ states of the world and 11 × 11 policy options for each time step yielding 3.10 × 10^37^ (11 × 11^18^) policy options between 2010 and 2100 (18 time steps of 5 years).

## Results

As outlined above, our analysis focused on two key thresholds, 350 and 550 ppm by 2100. Here we present the results of the analysis in terms of key attributes of the structure of the control variable and the most important state variables to track in order to remain viable. We will first address the 550 ppm goal followed by the more restrictive 350 ppm target.

### Carbon neutrality and staying below 550 ppm until 2100

We analyzed all combinations of the world states from 2010 and 2100 in order to characterize the adaptive policies, for each world state, that enable the planetary system to stay below 550 ppm until 2100. Our computations generated all viable states as a function of time. In order to represent this very large data set for 8 variables, we focus the analysis on the most influential variables for the viability of the system using Sobol indices (see Figs 2a–c and SI). As can be expected, the most significant variables for the viability of the system are the emission reduction rate *μ*(*t*) and the carbon stocks *M*_*AT*_(*t*) and *M*_*UP*_(*t*) in the atmosphere and in the upper part of the oceans. Therefore, it is necessary to closely monitor these stocks in order to derive adaptive climate policies with the objective of not crossing the planetary boundary. Note that the economic variable (the world capital stock) is the fourth most important variable (CO_2_ emissions are connected to the economy). The more we advance in time, the more the Sobol index for the atmospheric carbon stock increases (value triples between 2010 and 2060). This is the result of the fact that unlike the emission control rate, it is easier to control the carbon stock earlier than later. Moreover, the fact that the second order Sobol index 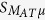 increases with time demonstrates that the emission control rate *μ* clearly depends on the amount of carbon *M*_*AT*_(*t*) in the atmosphere.

The set of viable states that complies with the planetary boundary of 550 ppm in 2010 is represented in Fig. 2d,e and f (red blocks) as a function of time and the three most important parameters with the maximum capacity of acting, 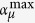, set to 1. The shape of the viability set (red blocks) is most strongly impacted by the emission reduction rate. But, for low values of the control rate, the shape of the viability kernel is more strongly influenced by carbon stocks. The minimum value of the emission reduction rate (for any state of the world) increases with time to 0.8 in 2060 (in the case of a value of the CO_2_ concentration in the atmosphere close to 550 ppm in 2060, see the lower boundary of the viability set in Fig. 2f). The main idea here is that even if the viable set is significant in the present, this set shrinks considerably (the volume of the red solid decreases) over time. However, the extent of this shrinking depends on the maximum capacity to act 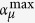. If we consider the viable states in 2010, our results illustrate a trade-off between immediate reduction and progressive reduction according to time (see Fig. 2g,h and i): we need an immediate emission reduction rate of around 33% for 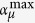 = 0.2 (see the lower boundary of the viability set on Fig. 2h). Therefore, regardless of future climate policies, considering an immediate massive reduction of CO_2_ emissions will enable a lower effort in the future. In Fig. 2i, the number of viable states (expressed in % according to the analysis set) that comply with the planetary boundary of 550 ppm is represented for different values of this capacity of acting 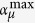. Obviously, the higher the capacity to act, the higher the viability of the system (see Fig. 2i). However, the difference between 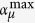 = 0.4 and 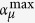 = 0.2 is more significant than the difference between 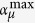 = 0.4 and 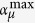 = 1. Therefore, the value of increased capacity to act is highly nonlinear and exhibits decreasing marginal returns – we should not overestimate the value of a large capacity to reduce emissions in the future relative to reducing emissions now. In addition, the viability of the system decreases until around 2075–2080; if the CO_2_ concentration is below 550 ppm around this time, it is then easier to maintain this concentration until 2100 because the peak atmospheric carbon concentration has been limited and the main emission reduction effort has already been done.

**Figure 1:**
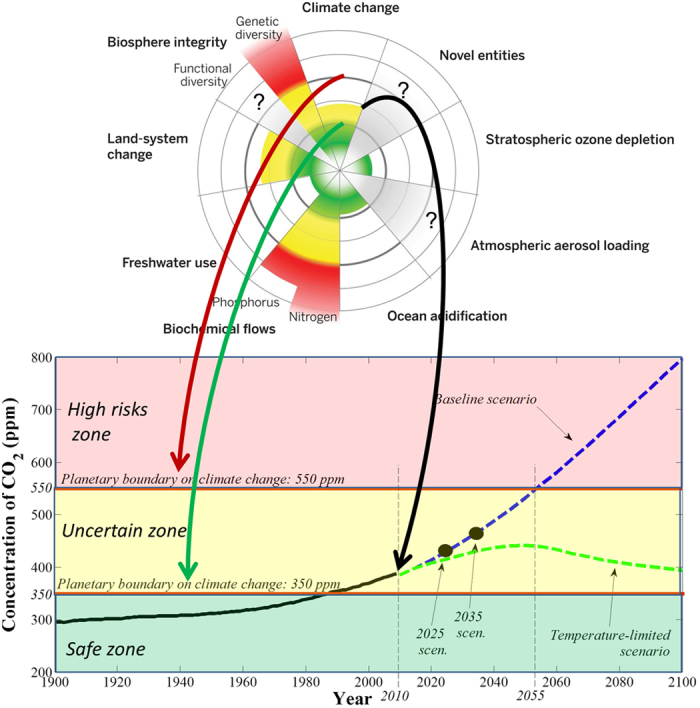
Climate change and planetary boundaries, inspired by Rockstrom *et al*.^1^,^2^. We use the planetary boundary^1^,^2^ on climate change: a CO_2_ concentration of 550 ppm as an objective for 2100 and a concentration of 350 ppm for 2100. The temperature-limited scenario, based on Copenhagen conference, complies with this planetary boundary. The “2010” scenario uses as initial point the baseline scenario in 2010. The “2025” and “2035” scenarios starts respectively in 2025 and in 2035 using the baseline scenario as initial conditions.

**Figure 2:**
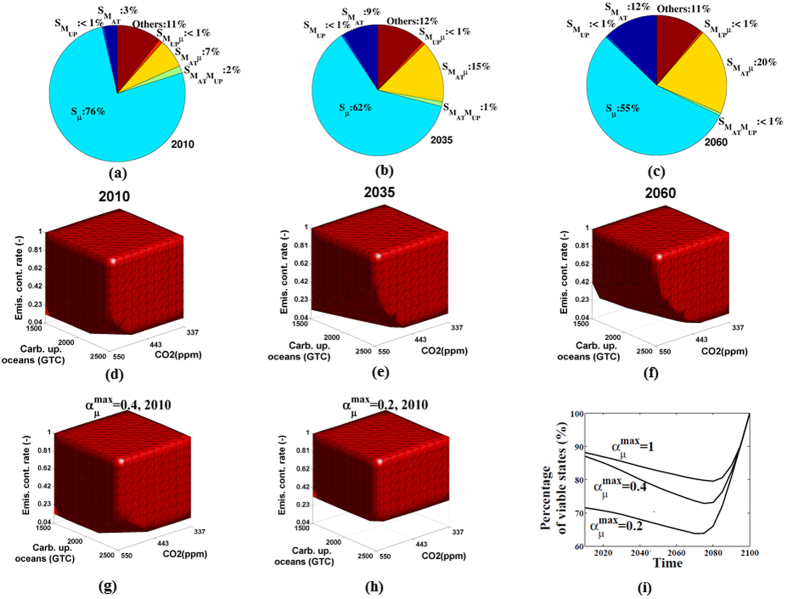
Viable states that comply with the planetary boundary of 550 ppm until 2100. Sobol indices (Figures **a**,**b** and **c** assess the most important state variables on the viability of the system. The more we advance in time, the more the carbon stock index increases (yellow region), unlike the emission control rate (blue region), because it is easier to control the carbon stock earlier than later. The most important variables (according to Sobol indices) are the emission control rate *μ*(*t*), the carbon stocks in the atmosphere *M*_*AT*_ and the carbon stocks in the upper oceans *M*_*UP*_. The viability kernels (red blocks) according to these variables are represented from Fig. 2d–f over time. We can see that the shape of the viability kernels is most strongly impacted by the emission control rate. But, for low values of the control rate, carbon stocks have more influence on the shape of the viability kernel. The capacity of reducing CO_2_ emissions, represented by 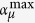, limits the viability of the system (see Fig. 2g,h and i).

**Figure 3:**
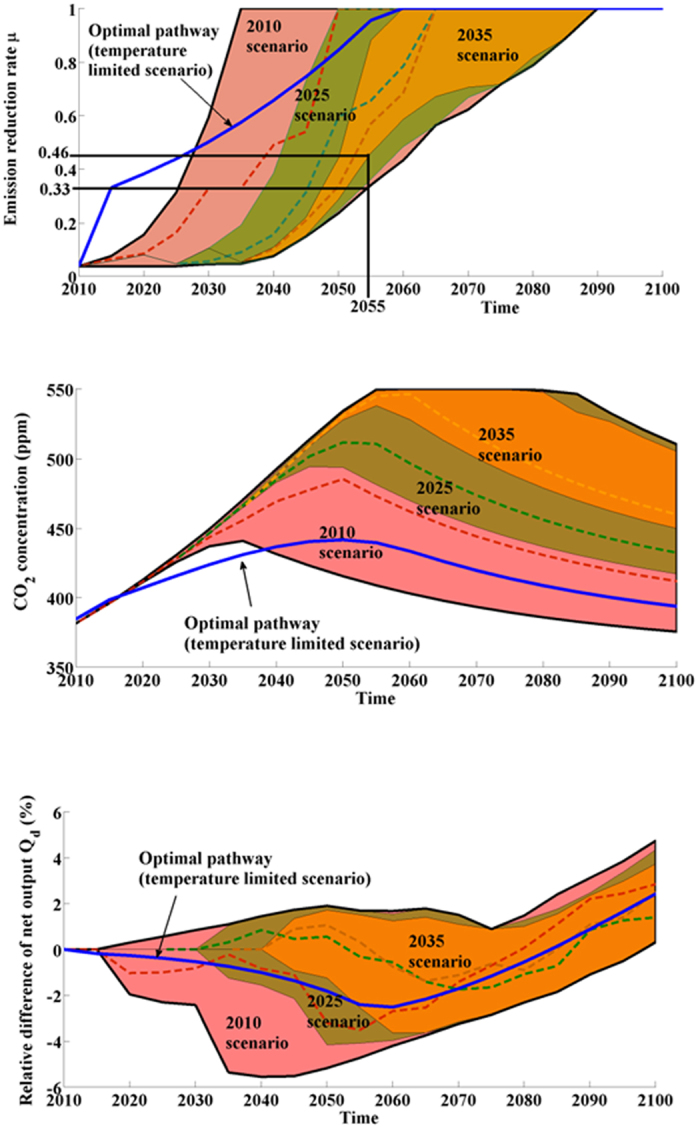
The impact of delayed policies on CO_2_ concentrations, emission reduction rate and relative GWP *Q*_*d*_. The set of trajectories of the 2035 scenario is included in the 2025 set which is included in the 2010 set. One random trajectory is also represented for each scenario. Note that the optimal pathway (from the temperature-limited scenario) expects an immediate reduction of CO_2_ emissions.

Although we have followed the baseline scenario for decades, governments are still struggling with a global agreement. It is thus natural to ask what the effect of delayed policies is. We use 2010 data as initial conditions in our analysis for assessing all possible pathways from the 2010 data (2010 scenario) and from two delayed policies scenarios (2025 and 2035 scenarios, see Fig. 1). Figure 3 represents all future possible states (using 10000 simulations) in terms of CO_2_ concentration, emission reduction rate and in terms of relative difference *Q*_*d*_(*t*) of GWP according to the gross world product of the baseline scenario *GWP*^*BS*^(*t*), defined as follows:


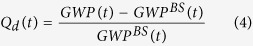


All scenarios consider carbon neutrality by 2090 unlike 2060 for the temperature-limited scenario and 2235 for the “‘baseline”’ scenario (see Fig. 3). For the 2010 scenario, it is necessary to reach an emission control rate equal to 33% (at least) in 2055, which constitutes a minimum milestone for future climate policy design. The CO_2_ concentration of these futures is not very close to the threshold of 550 ppm by 2100 because the main concern is to limit the carbon peak between 2050 and 2090. This peak can be reduced to 440 ppm (respectively 495 and 538 ppm) for the 2010 scenario (respectively for the 2025 and 2035 scenario). If effective policies are delayed, the peak will be very close to 550 ppm despite greater efforts. The mean increase of emission reduction rate is equal to 71% for the 2035 scenario (respectively 61% for the 2010 scenario) for a minimum CO_2_ concentration in 2100 around 455 ppm (respectively around 375 ppm for the 2010 scenario); therefore, efforts are greater for a worse result. In addition, the milestone “33% in 2055” becomes “46% in 2055” because of the lag in CO_2_ peak illustrating the importance of thinking of climate targets in terms of trajectories and not in terms of single points, the latter being meaningless in the case of lagged systems such as in the case of climate dynamics. Moreover, there is a risk that additional efforts will not be possible in practice because it is always more difficult to reach major agreements in order to provide significant interventions despite the emergency of the situation (see for instance Copenhagen climate talks in 2009). It is therefore essential to begin immediately, as delay will be costly in terms of achieving climate stabilization goals^35^,^36^. Note that if efficient climate policies are postponed after 2035, the planetary boundary will be crossed in all cases.

Our results show that estimations of GWP in 2100 are higher (for all scenarios) than the GWP in the baseline scenario because calculated trajectories rely on early total reduction of CO_2_ emissions (66% of the pathways exhibit a complete reduction of CO_2_ emission to zero in 2055 for the 2010 scenario), involving a relative decrease of GWP in the first years before an increase of this relative difference of GWP from 2060. It follows that the economy will grow faster than in the baseline scenario in the long term due to the benefits from avoiding climate change. As is almost always the case with the management of common-pool resources, during the transient period away from the inefficient trajectory (overuse) to the efficient trajectory, there is an immediate negative impact of use reduction (in our case an emission reduction yielding a lower CO_2_ concentration). Eventually, use reduction generates net benefits which, in our case, occur by 2100 (see SI, Figure S1 more for more details).

### Negative emissions and recovering 350 ppm in 2100

**Figure 4:**
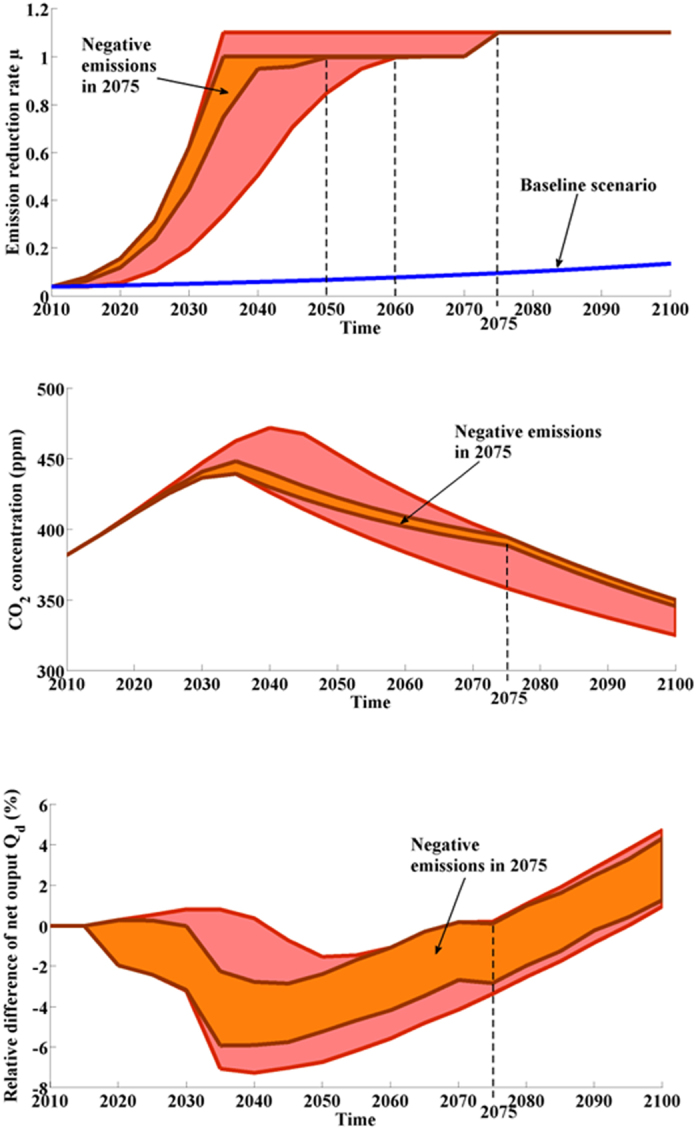
Recovering 350 ppm in 2100. Considering negative emissions (*μ*^max^ = 1.1 instead of 1) yields more flexibility and more options for reaching 350 ppm. However, if the implementation of climate engineering technologies is delayed to 2075 (orange area), carbon neutrality is necessary by 2050.

Having a concentration of 350 ppm corresponds to the climate planetary boundary considered as safe in terms of risks for the earth^1^,^2^. Many ecological systems have already experienced the effects of crossing this value, such as coral reefs, beyond which mortality increases^37^. Therefore, we study the states and the associated climate policies, for which the boundary of 350 ppm is not crossed. In comparison to the trajectories discussed above, there are none that enables the recovery of 350 ppm in 2100 from the 2010 initial conditions. We explored a doubling and tripling of the capacity of acting (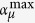 = 2 and 3) without success, even with carbon neutrality by 2025 (see SI, Figure S2, the “optimistic” case). Moreover, our results show (see SI, Figure S2) that doubling (or tripling) the capacity to act will provide a gain of around 20 ppm in 2100 because the climate lag makes it difficult to decrease CO_2_ concentrations in the atmosphere below 350 ppm. The maximum peak of CO_2_ is as important as the emission control rate^13^,^38^ as shown by the minimum peak that reaches 440 ppm in 2035 for 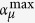 = 1 against 420 ppm in 2025 for 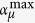 = 2 (see SI, Figure S2, blue clouds). To reach this objective, it has been shown^39^ that alternatives that rely on climate engineering in order to generate negative emissions^39^,^40^,^41^ are required. We therefore consider a scenario with negative emissions by allowing the emission reduction rate *μ*(*t*) to be higher than 1, i.e. we allow 10% negative emissions. This maximum limit for negative emissions is still being debated in the scientific community and estimates vary from 10 to 30 GtCO_2_/year^41^. We decided to be conservative by choosing 10% in order to allow for a maximum of negative emissions of around 10 GtCO_2_/year in 2100. The cost of negative emissions is a topic that is also still being debated (from $10 to $1000 tCO_2_^42^, see also Fuss *et al*.^43^ for more details). For our analysis, we assume that negative emissions have the same costs as abatement costs. Our results (see Fig. 4 done from 10000 simulations) show that it is necessary to achieve carbon neutrality and implement geoengineering technologies (*μ*(*t*) = 1.1) before 2075. After this date, it is not possible to bounce back to 350 ppm by 2100. Between 2045 and 2065, the relative difference of net output is negative in all cases since economic efforts are critical for complying with this planetary boundary by 2100. The date from which negative emissions are effectively introduced significantly influences the results. For instance, if we focus on the trajectories that consider negative emissions only from 2075, carbon neutrality has to be reached by 2050 (see Fig. 4, orange area). Indeed, delaying the introduction of such technologies after 2060 considerably reduces the number of viable pathways for recovering 350 ppm. Based on this analysis, access to technology that enables 10% negative emissions and reaching carbon neutrality in 2060 constitutes a clear milestone for the planetary boundary of 350 ppm in 2100. Moreover, so that this milestone will be relevant, it is necessary to get out of the baseline scenario by 2020 (see Fig. 4).

## Conclusion and Discussion

Although the sensitivity of our results to key assumptions on the implementation of geoengineering technologies (and to effective abatement and damage costs) should make us cautious about translating our quantitative results into decision-making process, this sensitivity can be used to help determine where we should invest in learning and gathering information. However, beyond our quantitative conclusions, our approach may contribute not only towards mapping out all possible options and all possible future trajectories but also towards investigating a wider set of views for designing climate policies for supporting both CO_2_ emission reduction and the development of breakthrough geoengineering technologies in order to keep the planet within the safe operating space. Table 1 summarizes the different windows of opportunity for each of the two planetary boundaries and highlights how they narrow over time. Although emission reduction efforts need to increase as climate policy is delayed, the main result identifies the requirement for carbon neutrality in 2090 (at the latest) for staying under 550 ppm. This objective is consistent with the Paris agreement whose objective is to achieve carbon neutrality during the second half of the 21^st^ century. With the recent ratification by the European Union, the Paris agreement enters into effect, making this objective of staying under 550 ppm credible. Recovering the safe planetary boundary of 350 ppm in 2100, on the other hand, requires such efforts in terms of CO_2_ emissions reduction that this is not a credible target if based only on the reduction of CO_2_ emissions. For bouncing back to 350 ppm in 2100, our results emphasize the urgency of the situation and the need to: (1) not delay efficient policies for navigating away from the baseline scenario; and (2) develop and implement geoengineering technologies enabling not only carbon neutrality, but 10% negative emissions before 2060. Reaching this objective of 10% of negative emissions requires the development of new strategies based on innovative geoengineering technologies that can be effective in the short term^44^. Whereas solar radiation management (SRM) may provide a very short-term solution for low cost cooling of the atmosphere, this technology may have a limited effect on ocean acidification^45^ and may lead to major political conflicts^39^. Carbon capture and storage (CSS)^46^,^47^ or carbon dioxide removal (CDR)^48^,^49^ technologies may decrease atmospheric CO_2_ concentration by sequestering current CO_2_ from the atmosphere^50^ but these technologies are expensive and are not effective today at the global scale. Moreover, even though CSS and CRD (and also SRM) are promising, we stress that geoengineering measures should not be considered as equivalent to CO_2_ emission reduction^51^ or as a political panacea that may delay efforts for reducing CO_2_ emissions^52^. Besides, a break in the growth of CO_2_ emissions is immediately required for facing climate challenge, and ocean acidification^53^,^54^. Finally, because planetary boundaries are entangled in complex ways it is necessary to consider interactions among planetary boundaries, e.g. biodiversity or phosphorous and nitrogen concentrations, in order to capture all the complexity of the dynamics of the planetary boundaries that cannot be reduced to those of climate change. Beyond these specific results we present here, it is necessary to derive adaptive indicators—such as those we propose in Table 1—in order to fulfill the need for monitoring for facilitating the design of climate policy^55^ and to face social and political uncertainties inherent to climate decision process. These indicators have to be dynamical and adaptive by using pathway-based targets instead of single point targets at a given time, the latter being meaningless in case of social or political delays.

## Additional Information

**How to cite this article:** Mathias, J.-D. *et al*. On our rapidly shrinking capacity to comply with the planetary boundaries on climate change. *Sci. Rep.* **7**, 42061; doi: 10.1038/srep42061 (2017).

**Publisher's note:** Springer Nature remains neutral with regard to jurisdictional claims in published maps and institutional affiliations.

## Supplementary Material

Supplementary Information

## Figures and Tables

**Table 1:** Main milestones for complying with the planetary boundaries on climate change in 2100.

	Milestones
550 ppm in 2100	
*2010 scenario*	33% of emission control rate in 2055
*2025 scenario*	35% in 2055 (+2%)
*2035 scenario*	46% in 2055 (+13%)
*Temperature limited scenario*	95% in 2055 (+62%)
350 ppm in 2100	
*Reference case (* 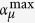 = 1)	No solution (375 ppm in 2100, optimistic case)
*Tripling efforts of emissions reduction (* 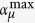 = 3)	No solution (355 ppm in 2100, optimistic case)
*Climate engineering (* 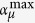 * = 1 and 0.1 of negative emissions)*	Carbon neutrality and effective geoengineering technologies are implemented before 2060
