# Supplementary material for: On our rapidly shrinking capacity to comply with the planetary boundaries on climate change
Source: Sci Rep. 2017 Feb 7;7:42061. doi: 10.1038/srep42061 (PMC5294404; doi:10.1038/srep42061)
Supplement: Supplementary Information [file srep42061-s1.doc]

##### Supplementary Information:

##### On our rapidly shrinking capacity to comply with the planetary boundaries on climate change

Jean-Denis Mathias^1^,[[1]](#footnote-2), John M. Anderies^2,3,4^, Marco A. Janssen^2,4^


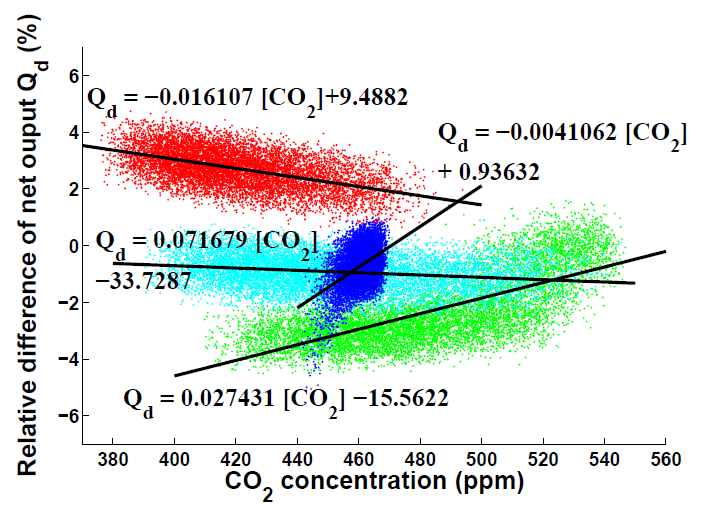

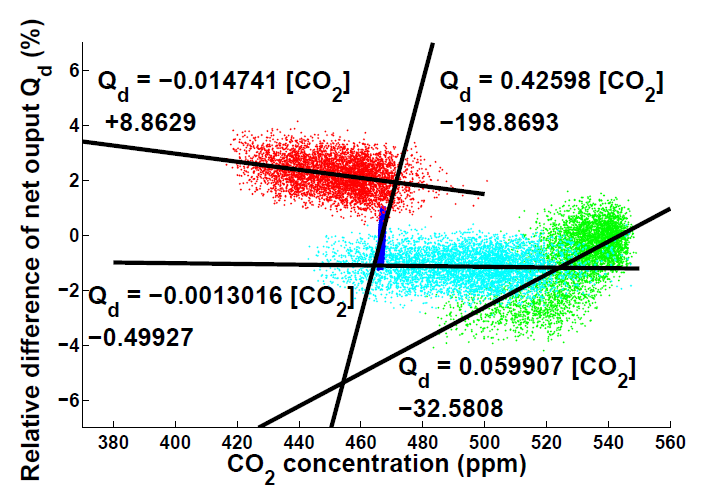

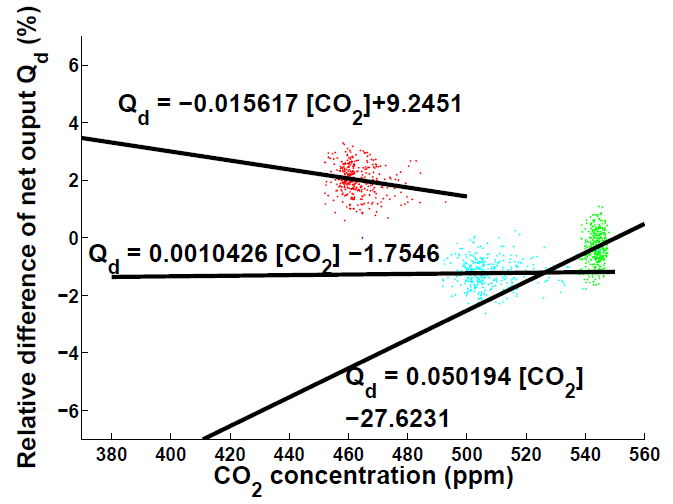


**2010 scenario 2025 scenario 2035 scenario**

**Supplementary Figure S1: Impact of delayed policies on relative GWP according to the GWP of the baseline scenario, CO_2_ concentration and emission control rate in 2035 (blue points), 2055 (green points), 2075 (cyan points) and 2100 (red points) from the 2010 initial states and for the 2010- 2025- and 2035- scenarios. The GWP is impacted in the first years in main cases but then the absence of climate damages will benefit to GWP until 2100. The slope between the relative difference of GWP and the CO_2_ concentration shows the trade-off between climate damages and abatement costs: the first years, implementing emission reduction policies has a negative impact on the GWP until 2075**

**Supplementary Figure S2: The impact of an increase of the acting capacity. We consider (optimistic scenario). Having (or ) instead of 1 (we double or triple the acting capacity) enables a decrease of 20ppm in 2100. Indeed, even with , the climate lag makes difficult the decrease of CO2 concentration in the atmosphere below 350ppm. The maximum peak of CO2 is as important as the emission control rate: the peak reaches 440ppm in 2035 for against 420ppm in 2025 for . Besides, as expected, the GWP is more impacted in the first years than in the case of staying below 550ppm and the main economic benefits of climate mitigation are delayed after 2075**

**DICE model (2013R)**

Main equations of the DICE (2013) model are recalled here. $E(t)$ is the CO2 emissions of carbon per year, composed of industrial emissions $E_{ind}(t)$ and emissions from land-use changes $E_{land}(t)$:

$E(t)=E_{ind}(t)+E_{land}(t)$ (3)

Emissions from land-use changes $E_{land}(t)$ writes:

$E_{land}(t)=E_{land}(t-1)(1-\delta_{E_{land}})$ (4)

Industrial emissions $E_{ind}(t)$ writes:

$E_{ind}(t)=\sigma(t)(1-\mu(t))A(t)K(t)^{\gamma}L(t)^{1-\gamma}$ (5)

$\sigma(t)$ is the estimate of the baseline carbon intensity:

$\sigma(t)=\sigma(t-1)[1+g_{\sigma}(t)]$ (6)

with:

$g_{\sigma}(t)=\frac{g_{\sigma}(t-1)}{1+\delta_{\sigma}}$ (7)

$\mu(t)$ corresponds to the emissions-reduction rate. The latter will constitute one of our controls (see next section "adaptive policy"). $A(t)$ constitutes the total factor productivity:

$A(t)=A(t-1)[1+g_{A}(t)]$ (8)

with:

$g_{A}(t)=\frac{g_{A}(t-1)}{1+\delta_{A}}$ (9)

The function $L(t)$ represents the population as well as the labor inputs. We take the trajectory of $L(t)$ with the UN medium trajectory (11 213 millions in 2100). The capital stock writes as:

$K(t)=I(t-1)Q(t-1)+(1-\delta_{k})K(t-1)$ (10)

$I(t-1)$ represent the reinvestment rate from the net economic output $Q(t-1)$. $I(t-1)$ represents a control (see section "‘adaptive policy"’). The global net output writes:

$Q(t)=\frac{1-\Lambda(t)}{1+\Omega(t)}A(t)K(t)^{\gamma}L(t)^{1-\gamma}$ (11)

The function $\Omega(t)$ represents climate damages:

$\Omega(t)=\varphi_{1}T_{AT}+\varphi_{2}T_{AT}^{2}$ (12)

$T_{AT}$ represents the mean surface temperature. The function $\Lambda(t)$ represents the abatement costs:

$\Lambda(t)=\theta_{1}(t)\mu(t)^{\theta_{2}}$ (13)

The abatment cost function coefficient $\theta_{1}(t)$ writes:

$\theta_{1}(t)=\frac{B^{p}(t)\sigma(t)}{1000\theta_{2}}$ (14)

with the backstop price (1000$ per tons of CO2):

$B^{p}(t)=B^{p}(t-1)(1-\delta_{B})$ (15)

The atmospheric temperature $T_{AT}(t)$ (degree celsius above 1900) and the deep temperature of oceans $T_{LO}(t)$ interact as follows:

$T_{AT}(t)=T_{AT}(t-1)+\xi_{1}[F(t)-\xi_{2}T_{AT}(t-1)-\xi_{3}(T_{AT}(t-1)-T_{LO}(t-1))]$ (16)

$T_{LO}(t)=T_{LO}(t-1)+\xi_{4}(T_{AT}(t-1)-T_{LO}(t-1))$ (17)

Variations in temperature depend on the change in total radiative forcings $F(t)$ of greenhouse gases since 1750:

$F(t)=\eta log_{2}(\frac{M_{AT}(t)}{M_{AT}(1750)})+F_{ext}(t)$ (18)

$F_{ext}(t)$ represents exogenous forcings:

$F_{ext}(t)=f^{2000}+0.01(f^{2100}-f^{2000})(t-2000)$ (19)

where $f^{2000}$ are 2000 forcings, non-CO2 GHG and influences and $f^{2100}$ are expected 2100 forcings, non-CO2 GHG and influences. Finally, the variables $M_{AT}(t),M_{UP}(t)$ and $M_{LO}(t)$ represent carbon in the atmosphere, carbon in a quickly mixing reservoir in the upper oceans and the biosphere, and carbon in the deep oceans. Carbon flows in both directions between adjacent reservoirs:

$M_{AT}(t)=E(t)+\varphi_{11}M_{AT}(t-1)+\varphi_{21}M_{UP}(t-1)$ (20)

$M_{UP}(t)=\varphi_{12}M_{AT}(t-1)+\varphi_{22}M_{UP}(t-1)+\varphi_{32}M_{LO}(t-1)$ (21)

$M_{LO}(t)=\varphi_{23}M_{UP}(t-1)+\varphi_{33}M_{LO}(t-1)$ (22)

Values of the parameters are described in Table S1. We consider $M_{LO}(t)$ as a forcing equation: simulations show that whatever the policy, it does not influence the carbon stock in deeper oceans at time horizon 2100.

**Sobol indices**

Sobol indices were calculated from the range analysis defined above. Considering a model *Y=f(X)*, first-order $S_{i}$ of variable $X_{i}$ writes as follows:

$S_{i}=\frac{Var\left(E\left[Y\left|X_{i}\right.\right]\right)}{Var(Y)}$ (26)

and the second-order indices of variables $X_{i}$ and $X_{j}$ are:

$S_{ij}=\frac{Var\left(E\left[Y\left|X_{i},X_{j}\right.\right]\right)-Var\left(E\left[Y\left|X_{i}\right.\right]\right)-Var\left(E\left[Y\left|X_{j}\right.\right]\right)}{Var(Y)}$ (27)

In our case, the variables $X_{i}$ are represented by the 6 state variables (carbon stocks and temperatures in the ocean and the atmosphere as well as the capital stock and the emission control rate) and the variable *Y* represents the viability of the system. The most important variables are the emission control rate $\mu(t)$, the carbon stocks in the atmosphere $M_{AT}$ and in the upper oceans $M_{UP}$. Then, then the viable sets of Figure 2 have been plotted through these variables and the **values of other dimensions correspond to the ones found in the baseline scenario**.

| **Variable** | **Type** | **Name** | **Unit** | **Value** |
| --- | --- | --- | --- | --- |
| $E(t)$ | Output | Total CO2 emissions | Giga tons of CO2 per year | $E(2010)$= 31.4 |
| $E_{land}(t)$ | Forcing equation | CO2 emissions due to land-use changes | Giga tons of CO2 per year | $E_{land}(2010)$= 1.54 |
| $\delta_{E_{land}}(t)$ | Parameter | Decrease of CO2 emissions due to land-use changes | per 5 year | 0.2 |
| $E_{ind}(t)$ | Output | Industrial CO2 emissions | Giga tons of CO2 per year | $E(2010)$= 29.860 |
| $\gamma$ | Parameter | Elasticity | - | 0.3 |
|  | Forcing equation | Estimate of baseline carbon intensity | tons of CO2 per 1000$ | = 0.489 |
| $g_{\sigma}(t)$ | Forcing equation | rate change of carbon intensity | % of change per year | $g_{\sigma}(2015)$= -1 |
| $\delta_{\sigma}$ | Parameter | parameter of $g_{\sigma}$ | % of change per 5 years | -0.1 |
| $\mu(t)$ | State variable | Emissions-reduction rate | % of change per 5 years | $\mu(2010)=3.9$ |
| $\alpha_{\mu}(t)$ | Control | Variation in emissions-reduction rate $\mu(t)$ | - | [0-$\alpha_{\mu}^{\max}$] |
| $A(t)$ | Forcing equation | Total factor productivity | - | $A(2010)$= 3.8 |
| $g_{A}(t)$ | Forcing equation | rate change of total factor productivity | % of change per 5 year | $g_{A}(2015)$= 7.9 |
| $\delta_{A}$ | Parameter | Parameter of $g_{A}$ | % of change per 5 years | 0.6 |
| $L(t)$ | Forcing equation | World population size | million | $L(2010)$= 6838 |
|  | State variable | World capital stock | trillion of 2005 $ | = 135 |
| $\delta_{k}$ | Parameter | Depreciation rate of the world capital stock | % of change per year | 10 |
|  | Output | Output net of damages and abatement | trillion of 2005 $ | - |
| $I(t)$ | Control | Reinvestment rate | - | [0.2366-0.2592] |
|  | Output | Climate damages | trillion of 2005 $ | - |
| $\varphi1$ | Parameter | Parameter of the climate damages function | - | 0 |
| $\varphi2$ | Parameter | Parameter of the climate damages function | - | 0.0027 |
|  | Output | Cost of climate damages | trillion of 2005 $ | - |
|  | Output | Abatement costs | trillion of 2005 $ | - |
| $\theta2$ | Parameter | Parameter of the abatement costs | - | 2.8 |
| $\theta_{1}(t)$ | Forcing equation | Abatement cost function coefficient | - | $\theta_{1}(2010)=0.060$ |
|  | Forcing equation | Backstop price | 1000 $ per ton of C02 $ |  |
|  | Output | Carbon price | per ton of C02 $ |  |
| $\delta_{B}$ | Parameter | Depreciation of the backstop price | per year | 0.025 |
| $F(t)$ | Output | Change in total radiative forcings of greenhouse gases since 1750 | Watts per square meter | $F(2010)$= 1.824 |
| $\eta$ | Parameter | Forcings at CO2 doubling | Watts per square meter | 3.8 |
| $F_{ext}(t)$ | Forcing equation | Exogenous forcings | Watts per square meter | $F(2010)$= 0.008 |
| $f_{1}$ | Parameter | 2000 forcings, non-CO2 GHG and influences | Watts per square meter | -0.06 |
| $f_{2}$ | Parameter | 2100 forcings, non-CO2 GHG and influences | Watts per square meter | 0.62 |
| $T_{AT}(t)$ | State variable | Atmospheric temperature | degree Celsius (above 1900) | $T_{AT}(2010)$= 0.83 |
| $T_{LO}(t)$ | State variable | Deep oceans temperature | degree Celsius (above 1900) | $T_{LO}(2010)$= 0.0068 |
| $\xi_{1}$ | Parameter | Parameter of the change in atmospheric temperature | - | 0.104 |
| $\xi_{2}$ | Parameter | Parameter of the change in atmospheric temperature | - | 1.1875 |
| $\xi_{3}$ | Parameter | Parameter of the change in atmospheric temperature | - | 0.088 |
| $\xi_{4}$ | Parameter | Parameter of the change in deep oceans temperature | - | 0.025 |
| $M_{AT}(t)$ | State variable | Atmospheric carbon stock | Giga tons of carbon | $M_{AT}(2010)$= 818.985 |
| $M_{UP}(t)$ | State variable | Upper ocean carbon stock | Giga tons of carbon | $M_{AT}(2010)$= 1527 |
| $M_{LO}(t)$ | State variable | Lower ocean carbon stock | Giga tons of carbon | $M_{AT}(2010)$= 10010 |
| $\varphi_{11}$ | Parameter | Parameter of the atmospheric carbon stock | per 5 years | 0.912 |
| $\varphi_{21}$ | Parameter | Parameter of the atmospheric carbon stock | per 5 years | 0.03833 |
| $\varphi_{12}$ | Parameter | Parameter of the upper ocean carbon stock | per 5 years | 0.088 |
| $\varphi_{22}$ | Parameter | Parameter of the upper ocean carbon stock | per 5 years | 0.95917 |
| $\varphi_{32}$ | Parameter | Parameter of the upper ocean carbon stock | per 5 years | 0.00034 |
| $\varphi_{23}$ | Parameter | Parameter of the deeper ocean carbon stock | per 5 years | 0.0025 |
| $\varphi_{33}$ | Parameter | Parameter of the deeper ocean carbon stock | per 5 years | 0.99966 |

**Table S1. Description of the variables used in the DICE model (2013R).** A state variable is a variable that is monitoring and used for designing policy through controls. The model parameters are the intrinsic variables of the DICE model. Forcing equations are exogenous dynamics that we cannot control. Outputs are functions needed for calculations of the state variables.

| **Variable** | **Type** | **Range of analysis** | **Range of viability calculation** | **Comments** |
| --- | --- | --- | --- | --- |
| $\mu(t)$ | State variable | [0.039-1] | [0.039-1] | Ranges are the same ( and $\mu(t)\leq1$) |
| $T_{AT}(t)$ | State variable | [0.8-4.2] | [0.8-4.2] | $T_{AT}^{e}q$ (atmosphere / oceans temperature) is between 1.3 and 3.2 (also matches the IPCC results) |
| $T_{LO}(t)$ | State variable | [0-0.9] | [0-1.8] | . Maximum of $T_{LO}(t)$=1.8 with the maximum values of the analysis range. |
| $K(t)$ | State variable | [135-1500] | [135-1640] | whatever the values of the parameters (within the analysis range). $K=1640$ is the maximum calculated value of $K$ in the analysis range (case of high population, high reinvestment and high capital stock). |
| $M_{AT}(t)$ | State variable | [819-1181] | [723-1181] | $M_{AT}(t)$ reaches 723 in the extreme case of no CO2 emission, during 90 years. The maximum value is the planetary boundary (constraint). |
| $M_{UP}(t)$ | State variable | [1527-2300] | [1527-2500] | on the analysis range. $M_{UP}(t)$=2500 in the case of $M_{UP}(t)$ and $M_{AT}(t)$equal the maximum value of the analysis range. |

**Table S2. Description of the variables used in the viability analysis**. The minimum and maximum values of the analysis range correspond to the minimum and maximum values from the baseline and the temperature-limited scenarios. The calculation range corresponds to the set of states necessary for calculate the viability of the states within the analysis range.

1. ^1^IRSTEA, UR LISC, 9 avenue des landais, 63170 Aubiere, France. ^2^School of Sustainability, Arizona State University, United States. ^3^School of Human Evolution and Social Change, Arizona State University, United States. ^4^Center for Behavior, Institutions and the Environment, Arizona State University, United States. Correspondence and requests for materials should be addressed to JDM (e-mail: jean-denis.mathias@irstea.fr) [↑](#footnote-ref-2)
